# Effect of doula support during labor on perineal and anal sphincter injury in primiparous women

**DOI:** 10.1007/s00404-025-08259-1

**Published:** 2026-01-13

**Authors:** Yoav Baruch, Uri Amikam, Emmanuel Attali, Ronen Gold, Asnat Groutz, Yariv Yogev, Nadav Michaan

**Affiliations:** Gray Faculty of Medical and Health Sciences, Lis Hospital for Women’s Health, Tel Aviv Sourasky Medical Center, Tel Aviv University, 6 Weizmann St, 6423906 Tel Aviv, Israel

**Keywords:** Doula support, Perineal injury, Perineal trauma, Obstetric anal sphincter injury (OASI), Primiparous, Vaginal delivery, Episiotomy, Vacuum extraction

## Abstract

**Purpose:**

To determine whether doula support during primiparous deliveries reduces the rate of perineal injury including obstetric anal sphincter injury (OASI).

**Methods:**

A retrospective cohort study was conducted at a single, tertiary university-affiliated medical center. This study included all primiparous women between the ages of 18 and 45 who underwent a vaginal delivery with a singleton pregnancy at term between January 2020 and January 2024. The study group consisted only of women with explicit documentation of doula presence during labor. The control group consisted of women with explicit documentation of no doula presence in the medical records. Maternal and obstetrical parameters were analyzed and compared between groups. Multivariate regression analysis was employed to assess factors associated with perineal injury.

**Results:**

Overall, 5866 primiparous women were included: 4,583 in the routine care group and 1283 with a doula support. Parturients in the doula group were older (32.5 vs. 30.7 years, *p* < 0.001) with lower rate of epidural analgesia (77.6% vs. 86.9%, *p* < 0.001). Women with doula support had slightly lower rates of overall perineal injury (91.2% vs. 92.9%, *p* = 0.041), a lower incidence of episiotomy (35.9% vs. 39.5%, *p* = 0.022), but no significant differences in the rates OASI (0.94% vs 1.1%, *p* = 0.630).

Multivariate regression analysis revealed that doula support was not associated with a significant reduction in perineal injury. Epidural use (OR 1.571, *p* < 0.001), high birthweight (OR 2.649, *p* < 0.001), and vacuum extraction (OR 6.736, *p* < 0.001) were identified as significant contributors to perineal injury.

**Conclusions:**

In primiparous births, doula support was not independently associated with lower overall perineal injury. Perineal protection likely depends on mechanical and clinical factors rather than continuous support alone.

## Take-home message

**Table:** 

In this cohort of 5866 primiparous births, doula support was not associated with a reduced incidence of perineal injury when controlling for mechanical factors. Perineal trauma was primarily driven by vacuum-assisted delivery, birth weight, and the use of epidural anesthesia.	

## Introduction

Perineal injury may arise from either episiotomy or spontaneous tears and can vary from small perineal or labial lacerations to more extensive injuries involving the anal sphincter complex. Perineal trauma remains a common complication of vaginal deliveries, particularly among primiparous women where the reported incidence of perineal injuries may be as high as 90% [1, 2]. Although a very common obstetric intervention in the past, a more restrictive use of episiotomies is now advocated, and as a result, episiotomy rate has steadily declined along the years [3–5]. Likewise, the implementation of national perineal protection programs led to a decline in the rates of obstetric anal sphincter injury (OASI) in the Western world [6, 7].

While the potential long-term consequences of severe injuries such as OASI are well established, even lower-grade perineal tears may carry meaningful implications, such as chronic pain, dyspareunia, and various forms of pelvic floor dysfunction [1, 4, 8]. Older reports as well as recent studies suggest that continuous labor support, such as the presence of a doula, may improve birth outcomes including shorter labor, reduced need for any analgesia during labor, and lower rates of both instrumental deliveries and cesarean deliveries [9–11]. Doula roles during labor include emotional support, providing a reassuring and calming presence that may reduce anxiety, physical support, such as advice on positioning, breathing techniques and massage and informational support that assists couples to understand labor stages and communicate with the medical staff in the unfamiliar labor and delivery environment. Moreover, their benefits extend beyond labor, being associated with a lower risk of postpartum depression [12–15]. Despite increasing recognition of the potential benefits of continuous labor support, the specific impact of doula involvement on the incidence and severity of perineal tears remains insufficiently understood. Current evidence is limited, leaving uncertainty regarding whether doula support meaningfully influences perineal outcomes in contemporary obstetric practice. The aim of this study was to determine whether doula support during primiparous deliveries reduces the rate of perineal injury.

## Methods

This was a retrospective cohort study conducted at a tertiary, university-affiliated medical center. The research protocol was approved by the local Institutional Review Board.

### Participants

Eligibility criteria included primiparous women aged 18–45 years with a singleton term pregnancy in vertex presentation who delivered at our institution between January 2020 and January 2024.

The study group consisted only of women with explicit documentation of doula presence during labor. The control group consisted of women with explicit documentation of no doula presence in the medical records. Excluded from the study were multiparous women, women delivered by cesarean sections and rare cases of non-cephalic deliveries. Participants included in the study were divided to two groups, deliveries with the presence of doula support during delivery and routine care.

### Routine care and doula support

Routine care referred to standard intrapartum management by a midwife, typically responsible for 1–3 delivery rooms simultaneously, with continuous bedside presence mainly during the second stage of labor. Doula support at our Labor and Delivery ward is permitted during labor and is used at the discretion of the parturient. The doula is not an official part of the obstetrical team, and as such, is not authorized to provide medical care or be part of the clinical decision-making during labor. Doulas do offer emotional as well as physical support as an additional component of obstetrical supervision. The presence of doula during labor was recorded in the electronic medical record.

### Data collection

All data were retrospectively extracted from complete electronic medical records. Data collected included: maternal demographics, intrapartum variables as well as obstetrical and neonatal outcomes. Data regarding spontaneous perineal tears, need for perineal repair, episiotomies, obstetric anal sphincter injury, length of hospital stay after delivery, and need for blood products transfusion were also recorded.

Maternal age, body mass index (BMI), gestational weight gain, and gestational age at delivery, induction of labor, prolonged second stage, analgesia type, episiotomy, and instrumental deliveries, mainly vacuum extraction, were considered potential confounders that might have influenced the rate of perineal tears. Episiotomies in our institution are mediolateral and performed selectively, at the discretion of the attending midwife or obstetrician, particularly in cases of operative vaginal delivery with vacuum extraction. Perineal tears were defined according to the ACOG’s classification system for perineal lacerations (1st degree: injury to the perineal skin and vaginal mucosa only; 2nd degree: injury extend deeper, involving the perineal muscles but not the anal sphincter; 3rd degree: injury to the anal sphincter complex; 4th degree; lacerations through the anal sphincter complex and the rectal mucosa) [16].

In cases where the same patient underwent an episiotomy and sustained an additional perineal or labial tear, both were documented separately. The rate of perineal lacerations, episiotomies and OASI rate was compared between groups.

### Sample size

Sample size was calculated using significance level of 5% and power of 80%. Approximately 10% of women are accompanied by doula during delivery at our Labor and Delivery ward. According to the literature we assume that among primiparous women, the rate of perineal trauma is 90% [1]. We assumed a possible reduction of 5% in the rate of perineal trauma in the presence of doula. Using these assumptions 3,800 deliveries were needed, of them 380 women accompanied by doula and 3,420 women without doula.

### Statistical analysis

Distribution of continuous variables was evaluated using histograms and quantile–quantile (Q–Q) plots. Normally distributed variables were reported as means and standard deviations. Continuous variables were compared using independent samples T test or Mann–Whitney test, according to distribution of variables. Categorical variables were described as frequencies and percentages and compared using chi-square test. Multiple imputation was used to overcome missingness in several predictor variables that otherwise limited the development of multivariate regression models. Multivariate logistic regression was used to study the association between the presence of doula during delivery and the need for surgical repair of perineal laceration while controlling for potential confounders. All statistical tests were two-sided and *p *value < 0.05 was considered statistically significant. SPSS software was used for all statistical analyses (IBM SPSS statistics, version 29.0.2, IBM Corporation, Armonk, New York, USA 2023).

### Bias

This retrospective cohort study is subject to several potential biases. Selection bias may exist as doula use was patient-initiated rather than randomized. We addressed confounding through multivariate regression adjusting for known risk factors. Information bias was minimized using standardized electronic documentation though doula support quality and duration were not captured. The retrospective design limits control for unmeasured confounders including specific perineal protection techniques and clinician experience.

### Ethics

This research was conducted in accordance with the principles of the Declaration of Helsinki and institutional ethical standards. This study was approved by the Institutional Review Board (approval no. TLV 0221–25). As this was a retrospective cohort study based on anonymized electronic medical records, informed consent was waived by the committee.

## Results

A total of 5866 primiparous women met the inclusion criteria, of whom 1283 (21.9%) received continuous support from a doula during labor and 4583 (78.1%) did not. The two groups were similar in most baseline characteristics (Table 1).

**Table 1:** Demographic and obstetrical characteristics of primiparous women delivered with or without doula support

	Routine care N = 4583	With doula N = 1283	*P*
Maternal age	30.71(± 4.29)	32.5(± 3.87)	** < 0.001**
Gestational age at delivery	39.78(± 1.07)	39.9(± 1.1)	**0.001**
Diabetes (all forms)	554 (12.1%)	145(11.3%)	0.442
Pre-pregnancy BMI (kg/m^2^)	22.28(± 3.83)	22.17(± 3.67)	0.666
Pregnancy weight gain (kg)	12.99(± 5.24)	12.84(± 4.86)	0.360
Maternal ethnicity:			**0.001**
Caucasian (*)	4465(97.4%)	1270(99.0%)	
Asians (*)	76(1.7%)	4(0.3%)	
African	42(0.9%)	9(0.7%)	
Induction of labor	1220(26.8%)	286(19.0%)	**0.002**
Occiput-Posterior Presentation	153(3.3%)	32(2.5%)	0.126
Meconium stain amniotic fluid	945(20.6%)	318(24.8%)	**0.002**
Epidural analgesia	3982(86.9%)	996(77.6%)	** < 0.001**
Prolonged second stage	441(9.6%)	165(12.9%)	**0.001**
Vacuum extraction	1084 (23.7%)	298 (23.2%)	0.751
Birthweight (g)	3220(± 383)	3238(± 378)	0.123
Apgar 1 < 7	160(3.5%)	47(3.7%)	0.764
Apgar 5 < 7	14(0.3%)	4(0.3%)	0.970
Uterine revision (for manual lysis or retained products of conception)	281(6.3%)	92(7.5%)	0.335
Prolonged length of stay (more than 72 h)	772(16.8%)	198(15.4%)	0.229
Readmission (within 6 weeks)	67(1.5%)	27(2.1%)	0.105

(*)Indicates statistical significance within ethnicity subgroups; BMI- Body mass index; OASI-Obstetric anal sphincter injury; NRFHR-Non-reassuring fetal heart rate monitoring; Significant values are shown in bold

Parturients, in the Doula group were slightly older (mean age 32.5 vs. 30.7 years, *p* < 0.001), with lower rates of labor induction (19.0% vs. 26.8%, *p* = 0.002) and less frequent use of epidural analgesia during labor (77.6% vs. 86.9%, *p* < 0.001). A higher proportion of women in the doula group had a prolonged second stage (12.9% vs. 9.6%, *p* = 0.001 (Table 1).

Perineal outcomes are presented in Table 2. Women with doula support were more likely to deliver with an intact perineum (8.8% vs. 7.1%, *p* = 0.041). The presence of a doula was associated with a reduction in the rate of episiotomy (35.9% vs. 39.5%, *p* = 0.022). No significant differences were observed between groups in the rates of spontaneous perineal or labial lacerations, or in the incidence of OASI (Table 2).

**Table 2:** Perineal lacerations, episiotomies and obstetrical anal sphincter injuries among primiparous women, with and without doula support

	Routine care N = 4583	With Doula N = 1283	*P*
Any perineal injury	4257(92.9%)	1170(91.2%)	0.041*
Episiotomy	1808 (39.5%)	461(35.9%)	0.022*
Spontaneous tears	2930(63.9%)	836(65.2%)	0.417
Labial tear	913(19.9%)	245(19.1%)	0.511
Perineal Laceration:	2726(59.5%)	793(61.8%)	0.122
1st degree	1070(39.2%)	305(38.4)	0.0953
2nd degree	1476(54.1%)	437(55.1%)	
1 or 2 not specified	130(4.8%)	40(5.0%)	
3rd degree	45(1.7%)	11(1.4%)	
4th degree	5(0.2%)	1(0.1%)	
OASI (3rd and 4th combined)	50(1.1%)	12(0.94%)	0.630

Some patients sustained both spontaneous tears (labial and/or perineal) and episiotomy, therefore, percentages may exceed 100%. Significant values are shown in bold

Multivariate logistic regression model is presented in Table 3. After multivariate adjustment, doula support did not remain a significant predictor (OR 0.83, 95% CI 0.66–1.06, *p* = 0.13). Significant predictors of perineal injury included vacuum-assisted delivery (OR 6.74, 95% CI 4.20–10.81, *p* < 0.001), neonatal birth weight (OR 1.102 for each 100 g of newborn weight, CI 1.07–1.14, *p* < 0.001), epidural analgesia (OR 1.57, 95% CI 1.23–2.00, *p* < 0.001), and occiput-posterior fetal position (OR 3.21, 95% CI 1.01–10.23, *p* = 0.049). Higher maternal BMI was also associated with a slightly increased risk of perineal injury (OR 1.033, 95% CI 1.003–1.064, *p* = 0.029). Other variables, including maternal age, gestational age, and prolonged second stage, were not independently associated with perineal injury.

**Table 3:** Multi-regression analysis of factors associated with the need for medical repair of perineal injury after first delivery

	OR	95% c.i	*P*
Presence of doula	0.834	0.660–1.055	0.130
Age (years)	0.987	0.964—1.012	0.309
BMI (kg/m^2^)	1.033	1.003—1.064	0.029*
Asian ethnicity	1.758	0.543—5.687	0.346
African ethnicity	0.677	0.260—1.762	0.425
Gestational age	1.059	0.958–1.171	0.262
Induction of labor	1.059	0.776—1.272	0.959
Occiput-posterior position	3.211	1.007—10.234	**0.049**
Prolonged second stage	1.000	0.661—1.513	0.999
Epidural analgesia	1.571	1.233—2.000	** < 0.001**
Vacuum extraction	6.736	4.197—10.810	** < 0.001**
Birthweight*	1.102	1.070–1.140	** < 0.001**

^*^Birthweight odds ratio is presented per 100-g increase in birthweight

Significant values (<0.05) are shown in bold

## Discussion

In this large cohort of primiparous women, we aimed to evaluate the association between doula support during labor and the risk of perineal injury, including episiotomy and spontaneous lacerations, as well as OASI. Our main findings were: 1) Doula assistance during labor was associated with a slightly lower risk of perineal injury, compared to women laboring without doula, although the absolute difference of 1.7% in the overall perineal injury is unlikely to be clinically meaningful and lost statistical significance after controlling for confounders. 2) The presence of doula did confer lower episiotomy rate. 3) The rate of OASI was similar between groups.

The strongest determinants of perineal trauma were obstetric factors, notably vacuum-assisted delivery, higher birth weight, use of epidural analgesia, and occiput-posterior fetal position, all well-established risk factors for perineal injury [2, 17]. Vacuum extraction, in particular, carried an adjusted odds ratio of 6.7 for lacerations requiring repair in our analysis, emphasizing the considerable trauma risk inherent to operative vaginal births. Epidural analgesia and persistent occiput-posterior positioning are known to increase the likelihood of prolonged second stage and operative intervention, which may explain their strong association with perineal lacerations. These findings align with the understanding that mechanical and clinical factors largely govern perineal outcomes, minimizing any direct protective effect of continuous labor support on perineal trauma.

In our cohort, the proportion of women with doula support was higher than anticipated. In accordance with previous data, only 7–8% of primiparas achieved an intact perineum after labor, reflecting the reality that the vast majority of women will experience perineal lacerations at first delivery. Still, it is important to remember that most lacerations usually heal well with minimal long-term dysfunction beyond the immediate recovery period compared to more severe perineal trauma [18–20].

Even though continuous labor support was not found to reduce the overall perineal injury rate, it does confer multiple benefits for maternal well-being that merit emphasis. It is still plausible that doula presence reduces anxiety and muscle tension but probably cannot moderate mechanical risk factors. In our study, women laboring with doulas had lower utilization of epidural analgesia, consistent with the idea that one-on-one support can improve pain coping and reduce reliance on pharmacologic pain relief. This finding aligns with prior evidence that doula-supported mothers utilize epidural analgesia less often and report less anxiety and greater satisfaction with their birth experience [11–15].

On the other hand, it is probable that women who plan to give birth without analgesia are more likely to seek the support of a doula. Likewise, episiotomy rate was significantly lower for women with continuous doula support, a finding that has not been previously described. The decision whether to perform an episiotomy is largely based on clinical experience of the obstetrician or the midwife, and as such, may be influenced by the presence of doula. These improvements in the subjective birth experience, while not captured by perineal injury rates, are clinically meaningful and the role of doulas should be viewed holistically, even if the reduction in physical trauma is subtle, if any, the emotional and experiential benefits for the mother can be significant.

Furthermore, our data show that the rate of OASI was not influenced by continuous doula presence and was lower than reported in the literature, particularly when considering that only primiparous women were included in our study. However, these rates were consistent with the low rates observed at our institution [1, 21]. The low OASI rate in our cohort may be related to local attributes, such as ethnicity, anatomy, birth weight, and obstetrical practices in our delivery rooms, all of which can impact OASI rate.

Our study has several strengths. It draws on data from a large cohort, extracted from elaborate electronic medical records. The availability of comprehensive labor and delivery parameters allowed adjustment for multiple potential confounders known to influence perineal laceration risk. The sample size substantially exceeded that required by our power analysis, enabling detection of even small differences between groups that could be clinically meaningful.

However, several limitations should be acknowledged. First, the retrospective design limits the ability to establish causality. Second, the presence of a doula was recorded as a binary variable, without assessment of the doula’s level of experience, quality of support or timing of involvement during labor. Third, the use of reported preventive measures designed to reduce the rate of perineal tears, such as warm vulvar compresses and perineal massage, was not recorded [22]. Fourth, the potential for selection bias, as women who intend to avoid epidural analgesia may be more inclined to hire a doula, which could partially influence observed associations.

Despite these limitations, the findings provide meaningful insight into the relationship between doula support and perineal outcomes and may help guide future research and clinical practice.

## Conclusions

While doula support during primiparous vaginal delivery may offer some benefits, their presence was not directly associated to reduced perineal injury. These findings emphasize the complexity of perineal injury prevention and suggest that more interventions, in conjunction with doula support, may be necessary to reduce perineal trauma requiring medical repair during labor.

## Data Availability

The data are available from the corresponding author upon reasonable request.
